# Repeated Low-Velocity Impact Properties of Hybrid Woven Composite Laminates

**DOI:** 10.3390/ma18204774

**Published:** 2025-10-18

**Authors:** Sawroj Mutsuddy, Deng’an Cai, Mohammed Hasibul Hossain, Xinwei Wang

**Affiliations:** State Key Laboratory of Mechanics and Control for Aerospace Structures, Nanjing University of Aeronautics and Astronautics, Nanjing 210016, China; sawroj@nuaa.edu.cn (S.M.); wangx@nuaa.edu.cn (X.W.)

**Keywords:** hybrid woven, composite laminates, low-velocity impact, repeated impacts, impact damage

## Abstract

Hybrid woven composite materials and structures have important application value in modern engineering because of their high specific stiffness, specific strength and excellent impact resistance. The mechanical properties of carbon/aramid fiber hybrid woven composite laminates under repeated low-velocity impacts were studied in this paper. This study aims to understand the behavior of these materials under repeated impact conditions and to evaluate their damage resistance and failure mechanisms. The materials and methods used are introduced in detail, including the preparation of samples, the experimental apparatus for impact testing, and the methods of damage assessment and data analysis. The experimental setup simulated real impact scenarios and followed procedures to collect and analyze data. The low-velocity impact tests were carried out in accordance with ASTM D7136 test standard. The experimental results show that with the increase in impact energy, the damage of laminates includes delamination, matrix cracking and fiber fracture. The damage threshold and damage propagation rate are affected by the type of fiber used and its lay-up direction in the composite. Compared with (0,90)_12_ laminates, [(0,90)/(±45)]_3s_ laminates show more obvious damage expansion, which highlights the importance of fiber orientation in the impact durability design of laminates. The results can be used to design and optimize the structure of hybrid woven composite laminates.

## 1. Introduction

Hybrid woven composite laminates, typically made from carbon and aramid fibers, are increasingly important in aerospace, automotive, defense, and sporting industries due to their unique combination of lightweight, high strength, stiffness, and impact resistance [1,2,3,4,5,6,7]. By combining carbon and aramid fibers, such laminates exploit the stiffness and tensile strength of carbon with the toughness and high impact resistance of aramid [8,9,10,11,12].

When combined, these fibers create laminates that balance brittle and ductile failure mechanisms, thereby improving damage tolerance and extending service life. This balance is especially critical for structures exposed to dynamic loads and potential impact events, where maintaining residual strength and preventing catastrophic failure are essential.

The mechanical response of composite laminates under low-velocity impact has been a major research focus due to the risk of hidden internal damage that significantly reduces structural performance. Baucom and Zikry [13] demonstrated that in woven laminates, damage typically initiates at the impact site with matrix cracking, which then propagates into fiber breakage and delamination. Kueh et al. [14] highlighted through their review that stacking sequence and laminate architecture strongly influence the resistance of composites to both single and repetitive low-velocity impacts. Lei et al. [15] showed that hybrid laminates reinforced with carbon and glass fibers possessed higher impact resistance and improved compression-after-impact strength compared to pure carbon fiber laminates. Similarly, Mathivanan and Jerald [16] and Sjöblom et al. [17] established key principles of low-velocity impact testing, confirming that woven architectures are highly sensitive to impact energy levels and often exhibit progressive internal damage rather than immediate catastrophic failure.

Since real service conditions often involve repeated or multiple impacts rather than a single strike, researchers have also examined fatigue-like impact behavior. Sadighi and Alderliesten [18] reviewed impact fatigue in fiber-reinforced composites, showing that repeated low-energy impacts cause gradual delamination growth and stiffness degradation. Liao et al. [19] investigated repeated low-velocity impacts in laminates and identified delamination thresholds, force plateaus, and proposed a damage index to characterize accumulation. Ekici et al. [20] extended these studies to SiC-reinforced aluminum metal-matrix composites, showing that repeated impacts could lead to strain hardening and improved toughness, underlining the complex nature of impact fatigue in different material systems. These studies emphasize that repeated impact introduces unique damage mechanisms compared to single impacts, including progressive matrix cracking, interfacial debonding, and delamination accumulation, which ultimately dictate fatigue endurance and structural safety.

This study aims to understand the behavior of carbon/aramid fiber hybrid woven composite laminates under repeated impact conditions and to evaluate their damage resistance and failure mechanisms. The materials and methods used are introduced in detail, including the preparation of samples, the experimental apparatus for impact testing, and the methods of damage assessment and data analysis. Therefore, this study investigates the repeated low-velocity impact performance of carbon/aramid woven laminates at energy levels of 8 J, 10 J and 12 J, focusing on endurance, damage evolution, force–displacement, force–time, energy–time responses and C-scan analysis. The findings provide valuable insights into the relationship between stacking sequence, damage mechanisms and impact resistance, offering guidance for the design of lightweight, damage-tolerant hybrid composites. The results can be used for engineers to design and optimize the structure of hybrid woven composite laminates.

## 2. Materials and Methods

### 2.1. Materials

**Figure 1:**
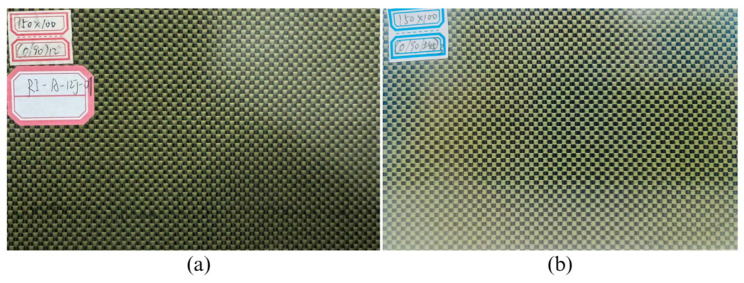
Specimens: (**a**) A with stacking sequences of (0,90)_12_; (**b**) B with stacking sequences of [(0,90)/(±45)]_3s_.

This study employs hybrid woven composite laminates used in this study were manufactured from carbon fiber (T300-3K, TORAY Inc., Tokyo, Japan) and aramid fiber (Kevlar^®^ 29, DuPont Inc., Wilmington, DE, USA), selected for their outstanding mechanical strength and high impact resistance. These reinforcements were combined with an epoxy resin system (MT0928, Weihai Hezong New Materials Technology, Co., Ltd., Weihai, China), which ensured effective fiber binding and enhanced the structural integrity of the laminates during the hot-pressing process. The laminates were fabricated from carbon/aramid fabric prepregs using the hot-press technique with the volume content of fiber of 57%. Each laminate consisted of 12 plies with an average dimension of 150 mm × 100 mm × 3.42 mm. Specimen A exhibits a repeated arrangement of fibers orientated at 0 (carbon fiber direction) and 90 (aramid fiber direction) degrees with respect to the loading direction, spanning over all 12 layers denoted as (0,90)_12_. In contrast, specimen B utilizes a more intricate stacking pattern of [(0,90)/(±45)]_3s_, with the addition of layers at ±45 degrees. This structure is specifically engineered to optimize the shear characteristics of the laminate and boost its overall impact resistance by efficiently distributing the impact energy across different fiber orientations. Specimens A and B are as shown in Figure 1.

Eighteen standard test specimens were manufactured. The specimens were divided into two groups, with each contained nine specimens. The impact energy was set to 8 J, 10 J, and 12 J. For convenience in discussion, the test pieces were named by the group name- repeated test time (01-03), the group name-impact energy- repeated test time (01-03) or the group name-impact energy- the time of repeated test (01-03)—the number of final impact to cause perforation. For example, A-8J-03-59th impact means that the group name is A, impact energy is 8 J, the time of repeated test impacted by 8 J energy is 03, and the number of final impact to cause perforation is 59. The meaning of other two names is similar and thus the definition is omitted. Every specimen was exposed to multiple impacts until a hole that penetrated completely through the material was discovered, indicating total failure of the material. To ensure the reliability and validity of the experimental findings, the test was conducted three times for each energy level on both specimen A and specimen B. Table 1 provides detailed mechanical properties of a specific composite material.

In Table 1, *E*_11_ and *E*_22_ denote the effective longitudinal and transverse elastic moduli of the laminate, while *G*_12_ refers to the in-plane shear modulus. The Poisson’s ratios, *µ*_12_ and *µ*_21_, describe the transverse strain response in the laminate when subjected to uniaxial loading in the longitudinal or transverse direction, respectively. The strength values are defined as follows: *X_T_* and *Y_T_* correspond to the tensile strengths in the longitudinal and transverse directions, *X_C_* and *Y_C_* represent the compressive strengths in those directions, and *S*_12_ indicates the in-plane shear strength.

Table 2 lists typical ply level properties of the individual carbon (T300-3K) and aramid (Kevlar^®^ 29) fibers. These values illustrate the contribution of each fiber constituent and contextualize the laminate-level properties reported in Table 1.

### 2.2. Repeated Low-Velocity Impact Tests

**Figure 2:**
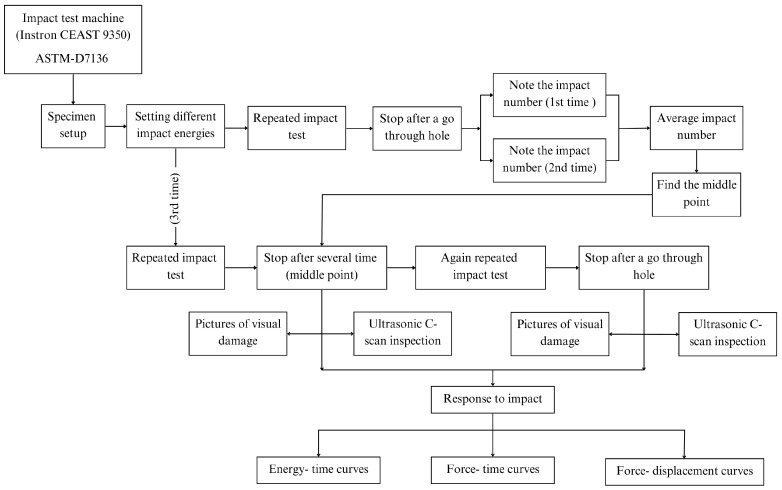
Flowchart of repeated low-velocity impact tests.

Figure 2 shows the flowchart of the repeated low-velocity impact tests. It includes establishing impact energies, carrying out impacts, stopping after penetration, recording impact numbers, and assessing visual damage and ultrasonic inspections in order to analyze impact responses using force-displacement, force-time, and energy-time curves.

**Figure 3:**
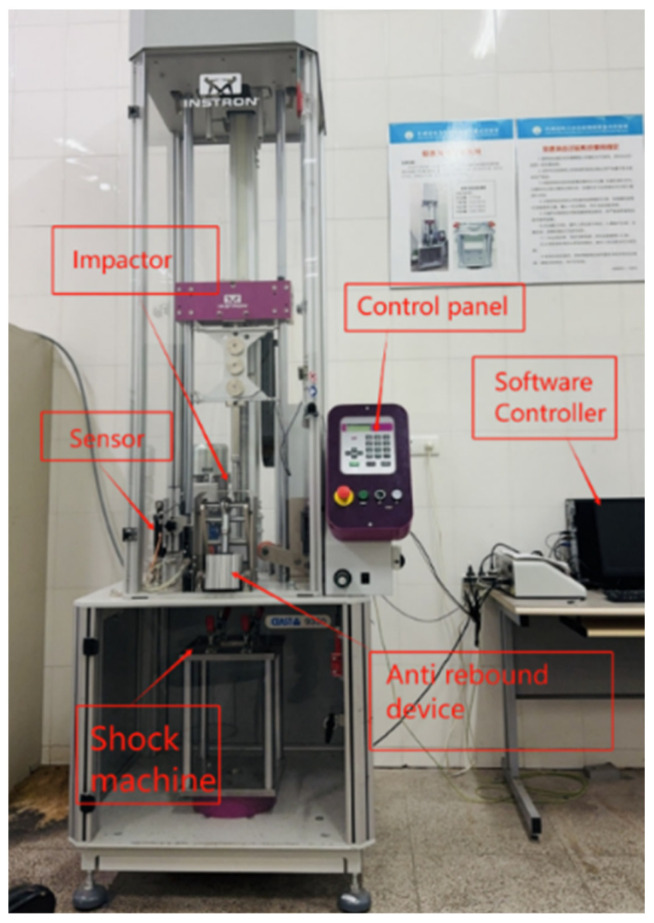
Impact test machine (Instron CEAST 9350).

Figure 3 shows the Instron CEAST 9350 drop-tower impact test machine (Instron Inc., Norwood, MA, USA) for repeated impact test. The major components are an impact tower, a controller, an acceleration system, and a test chamber. The acceleration system accelerates and lifts the impact tower with an air compressor. The impact tower includes a punch recovery/release system, an impactor, an impactor counterweight, an anti-rebound system, and impact and rebound optical velocity detectors to measure forces and rebound velocities during impact. In addition, the tester has an air compressor for hammer acceleration, a data collecting and processing system, and a software control system to ensure accurate test results. In real time, the data processing and acquisition system can record changes in the test specimen’s parameters such as energy, force, displacement, and time during the impact process. These data can be acquired to examine the test specimen’s performance and damage mode. In contrast, the impact device’s functioning and data collecting can be managed by the software control system via a computer, enabling automated testing and data processing.

The impactor consists of a hemispherical steel head with a diameter of 16 mm, connected to a steel round bar, and has a total mass of 3.277 kg. The impactor is aligned so that its axis remains perpendicular to the fixture plane throughout the testing process. The test specimen is placed on an impact support table, which has a rectangular through-hole measuring 125 mm × 75 mm. It is firmly held in place by four linkage mechanisms, each equipped with rubber-headed bolts, to ensure stable support during the impact test. The impact device allows for repeated low-velocity impact tests with varying impact energies by adjusting the height of the falling weight. To prevent damage from secondary impacts, an anti-rebound mechanism is integrated into the device. All tests were conducted in a room-temperature environment following the guidelines in the ASTM D7136 standard [21]. The specimens’ resistance to impact at various energy levels was evaluated using repeated impact tests with impact energies of 8 J, 10 J and 12 J. Every sample was exposed to multiple impacts until a hole that penetrated completely through the material was discovered, indicating total failure of the material. To ensure the reliability and validity of the experimental findings, the test was conducted three times for each energy level on both specimen A and specimen B, as was mentioned previously.

### 2.3. Ultrasonic C-Scan Device

**Figure 4:**
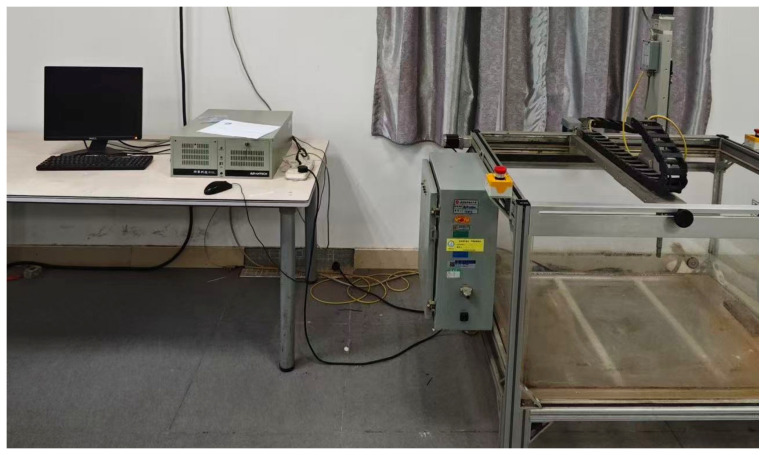
The UltraPAC immersion C-scan device.

Ultrasonic C-scanning was applied to assess the internal damage in the composite laminates after impact testing. The specimens were examined using an UltraPAC immersion C-scan system (Petroleum Analyzer Company, Houston, TX, USA) with 100 MHz frequency A/D transducer and 5 MHz ultrasonic probe, and the scanning rate is 0.5 mm/s, as illustrated in Figure 4.

## 3. Results and Discussion

### 3.1. Repeated Low-Velocity Impact Test Data Analysis

Table 3 shows how many impacts specimen A, stacking sequence is (0,90)_12_, survived under different energy levels before failing. At an 8 J energy level, the specimen displayed rather great endurance, requiring an average of 54 impacts to cause material failure. For 10 J impacts, resistance fell dramatically, with an average of 14 impacts across the three samples before failure. The specimen had the least endurance at 12 J, sustaining an average of only 9 impacts. Test data show that the specimen’s ability to absorb impacts decreases as the energy level increases, providing important insights regarding its structural limitations and durability under repeated stress conditions.

Table 4 displays the quantity of impacts needed to achieve full penetration, often known as “through-thickness” penetration, of specimen B with stacking sequence [(0,90)/(±45)]_3s_ at different energy levels. At an energy level of 8 J, specimen B exhibits significant resilience, necessitating an average of 106 impacts to accomplish penetration, with individual tests varying from 74 to 134 impacts. Nevertheless, as the energy level reaches 10 J, the average number of impacts decreases dramatically to 18, suggesting a notable decline in resistance to impacts with higher energy. At the maximum tested energy level of 12 J, the material’s endurance decreases even more, requiring an average of just 8 blows to penetrate the thickness. This clearly demonstrates the significant influence of higher energy levels on the strength and durability of the composite material. Test data highlight the crucial influence of the energy magnitude on the thresholds at which composite structures break.

**Figure 5:**
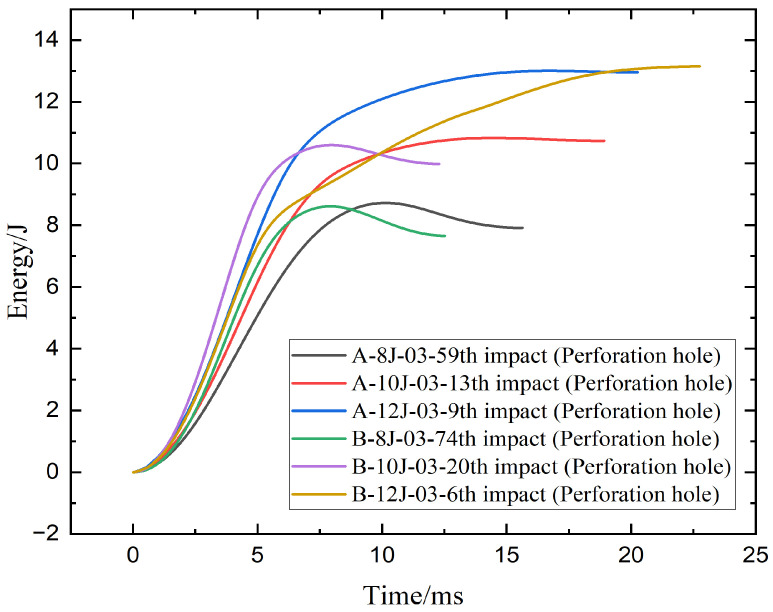
Energy absorption–time curves of specimen A and B under 8 J, 10 J, 12 J energies.

Figure 5 presents the energy–time curves for hybrid woven composite laminates under repeated low-velocity impacts at 8 J, 10 J, and 12 J energy levels. Each curve corresponds to the final impact that caused perforation. All specimens exhibit an initial rapid rise in absorbed energy, followed by a plateau indicating energy saturation and complete penetration. While the input energies were fixed, some curves slightly exceed these values due to system effects such as rebound or post-impact oscillations.

The energy-time response curves for specimens A and B at varying impact energy levels offer a clear picture of their post-perforation fatigue resistance and energy absorption properties. Both A-8J-03-59th and B-8J-03-74th show a plateau at 8 J at the time approximately 8 to 9 ms, indicating complete perforation. Specimen B tolerates more repeated impacts than specimen A (74 vs. 59), indicating superior fatigue resistance under low energy impact, even though specimen A absorbs slightly more energy (~8.2 J) than specimen B (~7.8 J). Compared to A-10J-03-13th, B-10J-03-20th exhibits a steeper initial rise and marginally higher peak energy at 10 J, suggesting a quicker and more thorough energy absorption process. Additionally, specimen B exhibits better durability as it withstands 20 impacts before failing, as opposed to 13 for specimen A. A-12J-03-9th, however, performs better at 12 J and can withstand 9 impacts. Both reach similar peak energies (~12.5–13 J), but the energy in specimen B builds more gradually over a longer time (~22 ms), implying more distributed absorption yet reduced fatigue resistance. Overall, specimen B shows enhanced performance at lower and moderate impact energies, while specimen A offers better impact resistance at higher energy levels.

**Figure 6:**
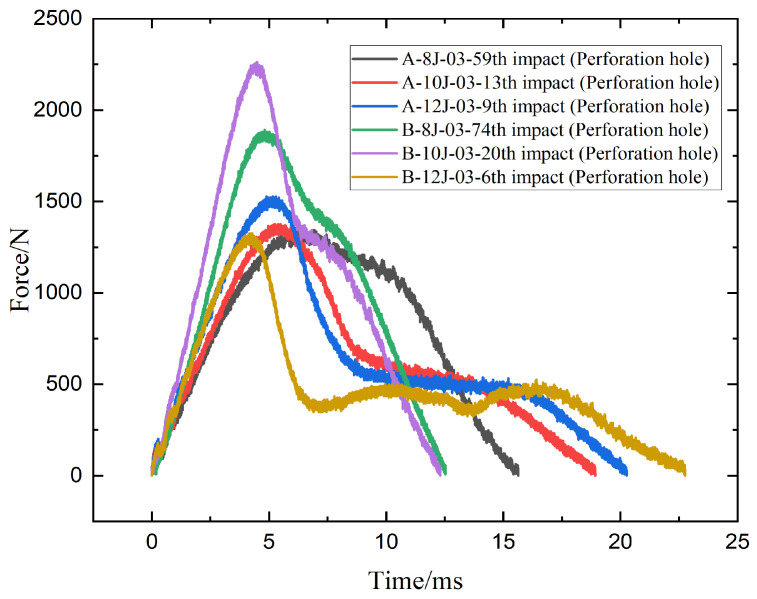
Contact force-time curves of the specimens A and B impacted by 8 J, 10 J, 12 J energies.

Figure 6 shows force-time response curves after perforation and critical differences in how specimens A and B behave under repeated low-velocity impacts at varying energy levels. At 8 J, both A-8J-03-59th and B-8J-03-74th exhibit a similar rising trend, but B-8J reaches a higher peak force (~1750 N) compared to A-8J (~1350 N), indicating a stiffer response prior to failure. This suggests that specimen B can resist higher loads during repeated impacts despite eventually failing at a later cycle (74th vs. 59th), reinforcing better fatigue resistance. At 10 J, B-10J-03-20th shows the highest peak force (~2300 N) among all curves, significantly exceeding A-10J-03-13th (~1500 N), highlighting superior load-bearing capacity and structural integrity of specimen B under moderate energy levels. However, at 12 J, the trend shifts, A-12J-03-9th maintains a peak force of around 1600 N, while B-12J-03-6th drops sharply to just ~1300 N with an overall flatter and noisier curve, indicating degraded structural performance and less efficient load transfer. This implies that at higher energy levels, specimen A outperforms specimen B in sustaining impact force. Overall speaking, specimen B demonstrates greater impact resistance at lower and moderate energy levels, while specimen A exhibits a more robust response under high-energy impact conditions.

**Figure 7:**
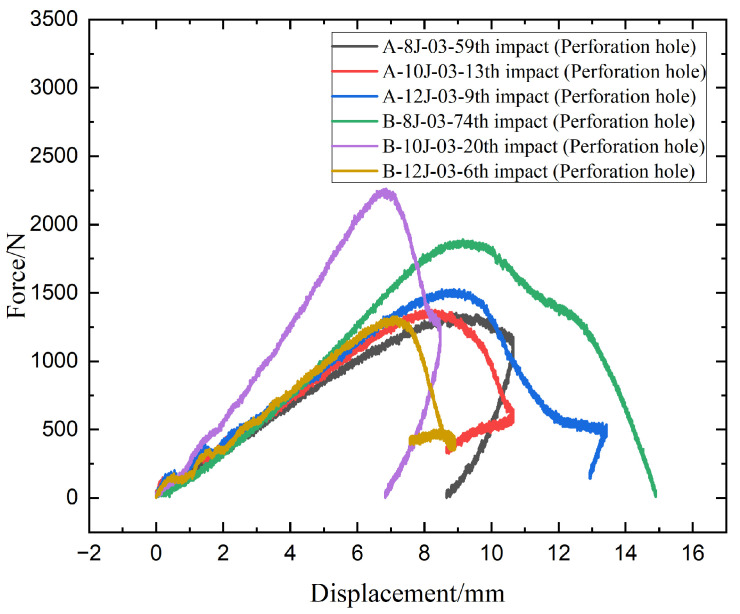
Contact force-displacement curves of the specimens A and B impacted by 8 J, 10 J, 12 J energies.

Figure 7 shows force-displacement response curves after perforation provide deeper insight into the structural behavior of specimens A and B under repeated low-velocity impacts. At 8 J, B-8J-03-74th displays both a higher peak force (~1800 N) and a larger maximum displacement (~15 mm) compared to A-8J-03-59th, whose peak load is around 1350 N at displacement of around 10 mm. This suggests that specimen B can endure higher loads and deform more before failure, reflecting greater ductility and energy dissipation capacity. At 10 J, B-10J-03-20th reaches the highest force (~2300 N) among all samples but fails suddenly at a displacement of only around 8 mm, indicating a more brittle failure despite high strength. In contrast, A-10J-03-13th shows moderate peak force (~1500 N) and higher displacement (~11 mm), suggesting a more balanced load-bearing and deformation behavior. At 12 J, A-12J-03-9th demonstrates a higher peak force (~1600 N) and larger displacement (~13 mm) than B-12J-03-6th, which shows a sharp drop in force beyond 8 mm displacement, indicating early failure and poor structural response. Overall speaking, specimen B exhibits greater stiffness and load bearing capacity at 8 J and 10 J, but specimen A offers better performance under 12 J impacts, with higher displacement before failure and less abrupt load drops, indicating superior toughness at higher energy levels. However, the peak force of B-12-03-6th drops significantly to about 1300 N and the displacement increases to 9 mm, showing rapid degradation. Specimen A performs better, maintaining higher force resistance over more impacts and showing less displacement increase compared to specimen B.

Overall speaking, specimen B demonstrates superior performance under low-energy impacts, such as 8 J and 10 J, exhibiting greater force resistance and durability. However, as the impact energy level increases to 12 J, specimen A outperforms specimen B by maintaining higher force resistance over more repeated impacts, indicating better durability and resistance to damage under higher energy impact conditions.

### 3.2. Ultrasonic C-Scan Results and Analysis

The UltraPAC ultrasonic C-scan imaging system was used to scan and inspect the specimens to obtain the damage condition of the specimens subjected to repeated low-velocity impact. Using ultrasonic C-scan imaging, the surface and internal structural damage condition and the material damage pattern of the specimens can be seen clearly. The Ultrasonic C-scan results are shown in Table 5, which provides detailed visual and quantitative information on the damage condition of specimens A and B in different energy levels, facilitating a deeper understanding of the material behavior under repeated impact conditions.

The C-scan results across all impact energies (8 J, 10 J and 12 J) reveal distinct differences in the damage evolution and endurance of specimen A and specimen B.

At 8 J, Specimen B demonstrated superior performance, sustaining up to 74 impacts before perforation, compared to 59 impacts for specimen A. The C-scan images confirmed this, as Specimen A displayed larger and more irregular delamination zones, while Specimen B maintained a more confined damage area despite the higher number of impacts.

At 10 J, the trend remained similar, with Specimen B again outperforming Specimen A by withstanding 20 impacts versus 13. The frontside C-scan for specimen A showed a large central dark zone indicating severe matrix cracking and delamination, while the backside revealed extensive damage spread. Specimen B, although showing slightly broader green and blue transition regions (reduced amplitude zones corresponding to partial delamination and matrix cracking), retained structural integrity for a greater number of impacts.

At the highest energy level of 12 J, however, the behavior shifted: specimen A endured 9 impacts before perforation, while specimen B failed earlier after only 6 impacts. The C-scan for Specimen A revealed a progressive failure pattern, with pronounced blue and green transition zones surrounding the penetration site, indicating gradual damage accumulation.

In contrast, specimen B exhibited a more abrupt failure with larger fully dark regions and fewer transition areas, reflecting a brittle-like collapse. Taken together, these results demonstrate that specimen B performs better at lower energies (8 J and 10 J), showing superior endurance and slower damage progression, whereas specimen A performs better at higher energy (12 J), where its damage evolution is more gradual and less catastrophic.

## 4. Conclusions

The mechanical properties of carbon/aramid fiber hybrid woven composite laminates under repeated low-velocity impacts up to perforating a through-thickness hole were experimentally investigated. The damage resistance and failure mechanisms are analyzed in detail. Based on the reported data, some conclusions may be drawn.

The results show that the hybrid woven composites exhibit distinct impact endurance behaviors depending on energy level: [(0,90)/(±45)]_3s_ laminates (specimen B) demonstrate superior resistance at low and moderate energies (8 J and 10 J), while (0,90)_12_ laminates (specimen A) maintain greater durability and load-bearing capacity at higher energy (12 J), reflecting the complementary contributions of carbon and aramid fibers to energy absorption and damage tolerance. The composites withstand repeated impacts before displaying symptoms of substantial damage. This feature is crucial for applications that materials require to withstand repeated stress without catastrophic breakdown. Specimen B demonstrates greater resistance and durability at energy levels below 10 J compared to specimen A, making it more suitable for lower-energy impacts. This enhanced resistance suggests that specimen B can better absorb and withstand impacts within this impact energy range. Conversely, specimen A shows a slight advantage at energy levels exceeding 10 J, indicating that it may be better optimized for withstanding higher-energy impacts. This differentiation in performance between the two kind of specimens suggests that (0,90)_12_ laminates may be more effective in scenarios involving extremely high energy impact. Layup [(0,90)/(±45)]_3s_ performs better than (0,90)_12_ in terms of both force resistance and deformation resistance, making it a more suitable choice for applications requiring high impact resistance and structural integrity.

As the impact energy increased, the laminates showed a progression of damage, including delamination, matrix cracking, and fiber breakage. The damage threshold and rate of damage progression are shown to be impacted by the type of fibers employed and their orientation inside the composite. [(0,90)/(±45)]_3s_ laminates exhibit distinct damage progression compared to (0,90)_12_ laminates, highlighting the importance of fiber orientation in impact durability.

## Figures and Tables

**Table 1:** Mechanical properties of the carbon/aramid hybrid plain woven laminate.

*E*_11_/GPa	*E*_22_/GPa	*G*_12_/GPa	*µ*_12_	*µ*_21_
51.24	19.74	2.64	0.093	0.32
*X_T_*/MPa	*Y_T_*/MPa	*X_C_*/MPa	*Y_C_*/MPa	*S*_12_/MPa
536.34	460.21	171.58	125.79	73.23

**Table 2:** Mechanical properties of T300-3K Carbon and Kevlar^®^ 29 Aramid Fibers.

Property	T300-3K Carbon Fiber	Kevlar^®^ 29 Aramid Fiber
Tensile Modulus (Longitudinal)	230 GPa	80 GPa
Tensile Strength (Ultimate)	3530 MPa	3000 MPa
Elongation at Break (Longitudinal)	1.5%	3.6%
Density	1.76 g/cm^3^	1.44 g/cm^3^

**Table 3:** Quantity of impacts for through-thickness hole of specimen A.

Energy	A-01	A-02	A-03	Average Number of Impacts
8 J	58	46	59	54
10 J	16	13	13	14
12 J	7	12	9	9

**Table 4:** Quantity of impacts for through-thickness hole of specimen B.

Energy	B-01	B-02	B-03	Average Number of Impacts
8 J	110	134	74	106
10 J	16	18	20	18
12 J	10	7	6	8

**Table 5:** Ultrasonic C-scan results of specimens after repeated low-velocity impact.

Specimen No.	Failure Stage (Through-Thickness Hole)			
	Top View		Bottom View	
A-8J-03	After 59th impact			
	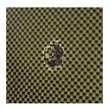	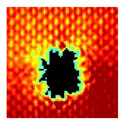	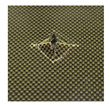	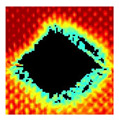
B-8J-03	After 74th impact			
	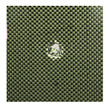	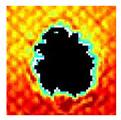	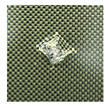	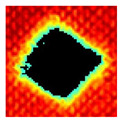
A-10J-03	After 13th impact			
	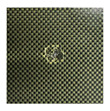	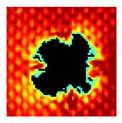	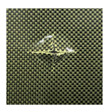	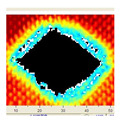
B-10J-03	After 20th impact			
	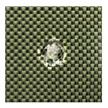	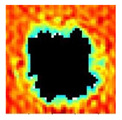	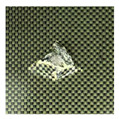	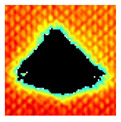
A-12J-03	After 9th impact			
	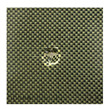	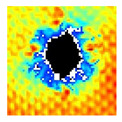	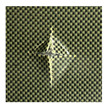	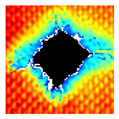
B-12J-03	After 6th impact			
	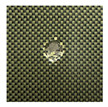	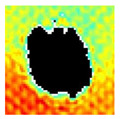	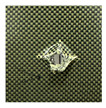	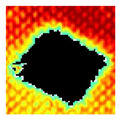
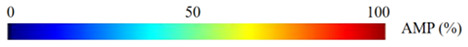				

## Data Availability

The original contributions presented in this study are included in the article. Further inquiries can be directed to the corresponding author.
